# Pineapple Vinegar Regulates Obesity-Related Genes and Alters the Gut Microbiota in High-Fat Diet (HFD) C57BL/6 Obese Mice

**DOI:** 10.1155/2020/1257962

**Published:** 2020-09-23

**Authors:** Nurul Elyani Mohamad, Swee Keong Yeap, Huynh Ky, Nancy Woan Charn Liew, Boon Kee Beh, Sook Yee Boo, Wan Yong Ho, Shaiful Adzni Sharifuddin, Kamariah Long, Noorjahan Banu Alitheen

**Affiliations:** ^1^Department of Cell and Molecular Biology, Faculty of Biotechnology and Biomolecular Science, Universiti Putra Malaysia, 43400 Serdang, Selangor, Malaysia; ^2^China-ASEAN College of Marine Sciences, Xiamen University Malaysia, 43900 Sepang, Selangor, Malaysia; ^3^College of Agriculture, Can Tho University, Can Tho, Vietnam; ^4^Institute of Bioscience, Universiti Putra Malaysia, 43400 Serdang, Selangor, Malaysia; ^5^Science Vision Sdn Bhd, Setia Alam, 40170 Shah Alam, Selangor, Malaysia; ^6^Faculty of Science and Engineering, University of Nottingham Malaysia, 43500 Semenyih, Selangor, Malaysia; ^7^Biotechnology Research Centre, Malaysian Agricultural Research and Development Institute (MARDI), 43400 Serdang, Selangor, Malaysia

## Abstract

Obesity is a pandemic metabolic syndrome with increasing incidences every year. Among the significant factors that lead to obesity, overconsumption of high-fat food in daily intake is always the main contributor. Functional foods have shown a positive effect on disease prevention and provide health benefits, including counteracting obesity problem. Vinegar is one of the fermented functional beverages that have been consumed for many years, and different types of vinegar showed different bioactivities and efficacies. In this study, we investigated the potential effects of pineapple vinegar as an antiobesity agent on a high-fat diet- (HFD-) induced C57BL/6 obese mice. C57BL/6 mice were treated with pineapple vinegar (1 mL/kg BW and 0.08 mL/kg BW) for 12 weeks after 24 weeks of HFD incubation. Serum biochemistry profiles, antioxidant assays, qPCR, proteome profiler, and 16S metagenomic were done posttreatment. Our data showed that a high concentration of pineapple vinegar (1 mL/kg BW) treatment significantly (*p* < 0.05) reduced the bodyweight (∼20%), restored lipid profiles, increased the antioxidant activities, and reduced the oxidative stress. Besides, significant (*p* < 0.05) regulation of several adipokines and inflammatory-related genes was recorded. Through the regulation of gut microbiota, we found a higher abundance of *Akkermansia muciniphila*, a microbiota reported to be associated with obesity in the high concentration of pineapple vinegar treatment. Collectively, these data established the mechanism of pineapple vinegar as antiobesity in mice and revealed the potential of pineapple vinegar as a functional food for obesity.

## 1. Introduction

Excessive consumption of food and an imbalanced diet has led to severe obesity issues in many people. As the food industry develops, the relative ease in procuring high-fat foods with low nutritional value has contributed to the increasing obesity cases [1, 2]. Furthermore, obesity is not an isolated case, but instead, it has a far-reaching impact on other diseases such as cancer and cardiovascular disease [3, 4]. Obesity is caused by the increase in adipocyte numbers and volume in the body [5, 6]. Adipocyte not only plays essential roles in fat storage but also involves in metabolic function and endocrine system of the body [7]. The alteration of adipose tissue leads to an increase of proinflammatory molecules and adipokines [8]. The relationship between obesity and inflammation has been studied for many years, and it was found that inflammation is one of the significant indicators linking to obesity [9, 10].

Functional food contains phytochemicals and bioactive compounds, which help to improve body function [11] and, consequently, alter the gut microbiota [12]. The use of functional food in treating obesity has long been investigated [13]. Fruits and vegetables which contain high antioxidants and minerals are examples of natural resources of functional foods [14–16]. Antioxidant has been proven to be one of the anti-inflammatory agents and plays a vital role in reducing weight through several mechanisms [17–19]. Studies showed that the consumption of fruit markedly decreased the risk of cardiovascular disease, which might be contributed by the presence of a high level of antioxidants and nutrients in the fruit [20–22]. The benefits conferred on the body by the consumption of fruits are at its peak when it is consumed fresh. However, fruits rot quickly in the tropical climate, especially when the supply exceeds the demand, and with improper handling, it causes an increase in fruit wastage worldwide, including in Malaysia [23–26]. To overcome this problem, fruits can be kept in several forms, including fermenting them into vinegar [25, 27].

Vinegar is one of the functional drinks that has been consumed not only for health maintenance but also to assist in the weight loss process [28, 29]. As vinegar can be made using various carbohydrate sources such as fruits, which is rich in antioxidants such as quercetin, it may assist in the weight loss process [30, 31]. A recent study on tomato vinegar has found that this vinegar exhibited *in vitro* and *in vivo* antiobesity effects [32, 33]. Tomato is rich with antioxidants and well known for its anti-inflammatory effect, which may be the factor that contributed to the antiobesity effect of tomato vinegar [34, 35]. In addition, coconut water vinegar and nipa vinegar were also reported with anti-inflammatory and antiobesity effects [36, 37].

Pineapple is one of the local fruits and is in abundance in Malaysia. It contains high vitamin C content and other antioxidants, which helps to enhance the immunity and protect the body from various diseases [38, 39]. It has been researched thoroughly and is reported to possess anti-inflammatory activity [40]. This is consistent with the data from our previous study, where antioxidant and anti-inflammatory activities have been shown by pineapple vinegar when tested *in vivo* [41]. As the bioactivities of vinegar were found to be varied due to the starting materials to prepare the vinegar [36], this study was conducted to investigate the antiobesity potential of pineapple vinegar using C57BL/6 mice fed with high-fat diets. Here, we performed several molecular assays and ran 16S metagenomic analysis to further explore the mechanism effect of pineapple vinegar in assisting the weight reduction. As far as we know, this is the first report on the antiobesity potential of pineapple vinegar tested *in vivo*.

## 2. Materials and Methods

### 2.1. Sample Preparation

Pineapple vinegar containing 6–8% of acetic acid was produced from pineapple fruits by 7–10 days of anaerobic fermentation with *S. cerevisiae* 7013 INRA (28–30°C) following four weeks of aerobic fermentation with *Acetobacter aceti* vat Europeans (28–30°C) [41].

### 2.2. Animals

Seven-week-old male C57BL/6 mice (*n* = 24) were obtained from Monash University Malaysia Campus, Malaysia. All mice were acclimatized for seven days and were given standard pellet diet and distilled water ad libitum. The mice were placed in plastic cages at room temperature (22 ± 1°C) with 12 h of dark/light cycle and relative humidity approximately 60%. This study was done according to the guidelines and was approved by the Institution Animal Care and Use Committee (IACUC), Universiti Putra Malaysia (UPM/IACUC/AUP-R097/2014).

### 2.3. Experimental Design

The study was done according to previous studies [36, 37]. The mice were divided into three groups, with eight mice for each group as follows:

  UT: induced mice were given HFD + distilled water (control)
  PL: induced mice were given HFD + 0.08 mL/kg BW pineapple vinegar
  PH: induced mice were given HFD + 1 mL/kg BW pineapple vinegar

A high-fat diet (HFD, D12451, 45% kcal fat, Research Diet, USA) was used to induce obesity for 24 weeks in all mice. In brief, low-dose (PL, 0.08 ml/kg bodyweight (BW)) and high-dose (PH, 2 ml/kg BW) groups were given pineapple vinegar daily via oral gavage, starting from week 24 until week 33. Untreated obese control mice (UT) were given distilled water only. The pineapple vinegar dose was selected based on the previous in vivo study of the antioxidant effect of pineapple vinegar [41]. Bodyweight was measured once a week. At the end of the treatment, mice were sacrificed using the cervical dislocation method. Blood, liver, and white adipose tissue (gonadal fat pad) were collected from the mice before further analysis.

### 2.4. Biochemistry Profile

Blood was collected from all mice, and serum was separated using the microtainers (BD, USA). Sera were diluted 10x and tested for cholesterol, triglycerol, LDL, and HDL using a biochemical analyzer (Hitachi 902 Automatic Analyzer) and adapted reagents from Roche (Germany).

### 2.5. Determination of Antioxidant Activities in Liver Homogenate

The following antioxidant activities of pineapple vinegar in liver homogenate were done according to the methods described in our previous study [41].

#### 2.5.1. FRAP Assay

The master solution consisted of acetate buffer, TPTZ, and FeCl_3_·6H_2_O solution at a ratio of 10 : 1 : 1 was prepared, wrapped in aluminium foil, and warmed to 37°C. Then, in a 96-well plate, 80 *μ*L of liver homogenate and 150 *μ*L of master solution were mixed and incubated for 10 minutes. The absorbance was read at 593 nm using an ELISA Plate Reader (Bio-Tek Instruments, USA). The activity was calculated from a standard FeSO_4_ calibration curve.

#### 2.5.2. SOD Assay

A serial dilution of liver homogenate (100 *μ*L/well) was prepared in a 96-well plate. 200 *μ*L of a master solution consisted of 0.1 mol/L phosphate buffer, 0.15 mg/mL sodium cyanide in 0.1 mol/L ethylene diamine tetraacetic acid (EDTA), and 1.5 mmol/L nitroblue tetrazolium, and 0.12 mmol/L riboflavin was then added to the plate before it was measured at 560 nm using an ELISA Plate Reader (Bio-Tek Instruments, USA).

#### 2.5.3. MDA Assay

200 *μ*L of liver homogenate was properly mixed with 800 *μ*L of PBS, 25 *μ*L of 8.8 mg/mL butylhydroxytoluene, and 500 *μ*L of 50% trichloroacetic acid. The mixture was incubated for 2 hours on ice and centrifuged (MX-160 Tomy, Japan) at 2000 rpm for 15 min. Then, 1 mL of the supernatant was collected and transferred into a new tube to be added with 75 *μ*L of 0.1 M EDTA and 250 *μ*L of 0.05 M 2-thiobarbituric acid. After that, the mixture was boiled for 15 min, and the absorbance was measured at 532 nm and 600 nm using an ELISA Plate Reader (Bio-Tek Instruments, USA).

#### 2.5.4. NO Assay

The assay was done following the manufacturer's protocols for Griess reagent assay (Invitrogen, USA). In brief, 150 *μ*L of liver homogenate was mixed with 20 *μ*L of Griess reagent and 130 *μ*L of distilled water in a 96-well plate. The mixture was incubated for 30 minutes and was measured at 540 nm using an ELISA Plate Reader (Bio-Tek Instruments, USA).

### 2.6. Gene Expression Analysis Using qRT-PCR

The mRNA expression of liver tissue was determined using real-time PCR following the methods described in the previous study [41]. In brief, the mRNA from the gonadal fat pad was extracted using the RNeasy Mini Kit (Qiagen, Germany). 1 *μ*g of the total RNA was then reverse-transcribed to the first-strand cDNA using iScript™ Reverse Transcription Supermix for RT-qPCR (Bio-Rad, USA) according to the manufacturer's protocols. Quantitative real-time PCR was performed with iTaq™ Universal SYBR® Green Supermix (Bio-Rad, USA). The quantities of the target genes and the housekeeping genes were calculated according to a standard curve, and the expressions were measured using iQ5 Software (Bio-Rad, USA). The expression levels in all samples were compared with those in the untreated group, and the levels of different mRNAs in the untreated group were designated as 1. All results were expressed as fold changes and measured in triplicate. Nontemplate controls were used to confirm specificity. The primer sequences used in the study are given in Table 1.

### 2.7. Proteome Profiler Assay (Adipokine)

The effect of pineapple vinegar on secreted cytokine from adipose tissue was analyzed using an adipokine proteome profiler kit (R&D System, USA). In brief, the membranes were blocked using blocking buffer at room temperature for 1 hour. Concurrently, the protein samples were incubated with the detection antibody at room temperature for 1 hour. Next, the protein samples, coupled with the detection antibody, were added to the membrane and incubated at 4°C overnight. The next day, the membranes were washed three times using a washing buffer and incubated with streptavidin-HRP for 30 minutes. Lastly, the membranes were rewashed three times before the substrate was added to develop chemiluminescence conditions. The membrane was then viewed using ChemiDoc XRS (Bio-Rad).

### 2.8. Metagenomic Study

Only untreated and high-concentration treatment groups were selected to undergo the metagenomic analysis. Faeces from week 20 (end treatment) were collected and kept at −80°C until further use. On a respected day, the faeces from each group were mashed using liquid nitrogen in an overnight baked pestle. The faeces were weighed, and the DNA was extracted using the QIAamp DNA stool mini kit (QIAGEN, Germany) according to the manufacturer's protocol. The DNA was purified using Agencourt® AMPure® XP beads (Beckman Coulter, USA), and the concentration was measured using a Qubit fluorometer (Life Technologies, USA).

Amplification of 16S amplicon of V3 and V4 regions was done in the first PCR (forward primer:5′-CGTCGGCAGCGTCAGATGTGTATAAGAGACAGCCTACGGGNGGCWGC AG-3′; reverse primer:5′-GTCTCGTGGGCTCGGAGATGTGTATAAGAGACAGGACTACHVGGGTATCTAATCC-3′). In brief, 25 *μ*l of DNA samples with a concentration of 12.5 ng of DNA, 12.5 *μ*l of 2x KAPA Hifi Hot Start Ready Mix (Kapa Biosystems), and 1 *μ*M of 16S amplicon PCR forward and reverse primer were mixed. The PCR was set to 95°C for 3 min, followed by 35 cycles for 30 s at 95°C, 30 s at 55°C, and 30 s at 72°C. The quality check for the first PCR product was done by purifying the product using Agencourt® AMPure® XP beads (Beckman Coulter, USA) before running them with the agarose gel to confirm the correct size. The second PCR will be commenced once all samples pass the quality check. The second PCR amplification was done by adding the product from the first PCR with the unique barcode using the Nextera XT index kit (Illumina, USA). In the second PCR amplification, 5 *μ*l from the first PCR products, 25 *μ*l of 2x KAPA Hifi Hot Start Ready Mix, and Nextera XT index Primers 1 and 2 were mixed. PCR was set to 95°C for 3 min, followed by eight cycles of 30 s at 95°C, 30 s at 55°C, and 30 s at 72°C and finally 5 min at 72°C. The quality check for the second PCR product was done using Agencourt® AMPure® XP beads and Agilent Bioanalyzer 2100 (Agilent Technologies, USA). Based on the bioanalyzer result, the concentration of the products was normalized to 4 nM and pooled together. Pooled samples were then prepared using the standard library protocols to a final concentration of 9 pM and ran on the MiSeq Sequencer (2 × 300 bp paired-end reads) (Illumina) using 600 cycles MiSeq V3 reagents (Illumina). The 16S metagenomics analysis was performed using Megan (MEtaGenome ANalyzer) based on BLASTX comparison against the NCBI database.

### 2.9. Statistical Analysis

All quantitative measurements were conveyed as mean ± standard deviation (SD). The analysis was performed using a one-way analysis of variance (ANOVA), and the group means were compared by the Duncan test. *p* values <0.05 were considered as statistically significant.

## 3. Results

### 3.1. Pineapple Vinegar Reduces Bodyweight

**Figure 1:**
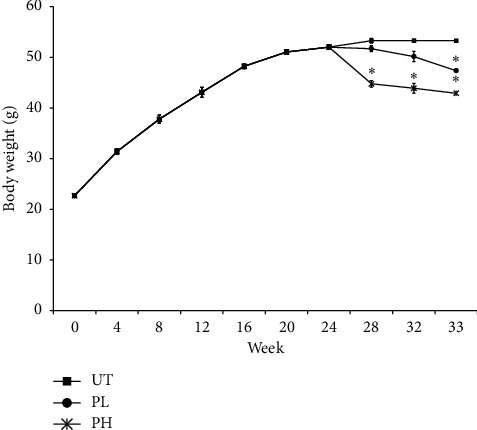
Bodyweight measurement (week 0–week 33) of untreated (UT), 0.08 mL/kg BW pineapple vinegar (PL), and 1 mL/kg BW pineapple vinegar (PH). The data presented are representative of the average biological replicate of mice from the same treatment group.

Figure 1 shows that the consumption of a high-fat diet for 24 weeks led to an increase in the bodyweight of mice in all groups. As the treatment began, we can see that the weight of the mice in the untreated group continues to increase, while weight loss reduction was noticed in both pineapple vinegar treatment groups. The posttreatment assays revealed the significant (*p* < 0.05) reduction in the percentage of gonadal adipose tissue over the bodyweight was recorded in the mice of high-concentration pineapple vinegar group as compared to the untreated group (Table 2).

### 3.2. Pineapple Vinegar Restores Lipid Serum Marker Enzyme

The effects of a high-fat diet and pineapple vinegar treatment in serum biomarkers are shown in Table 3. The serum level of cholesterol and triglyceride was the highest in the untreated group (G1), while the levels reduced significantly (*p* < 0.05) in the pineapple vinegar treatment groups. Besides, significant (*p* < 0.05) improvements were noted in the level of HDL/LDL ratio in the pineapple treatment groups compared to the untreated group.

### 3.3. Pineapple Vinegar Increases Antioxidant Activities

**Figure 2:**
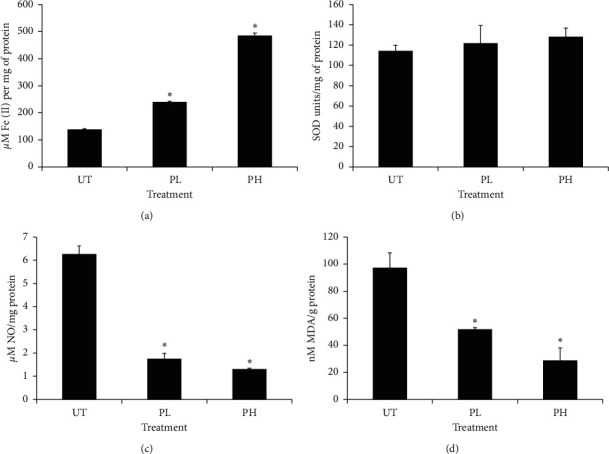
The antioxidant profile of untreated (UT), 0.08 mL/kg BW pineapple vinegar (PL), and 1 mL/kg BW pineapple vinegar (PH). The data presented are represented as mean ± SD of biological replicate of mice from the same treatment group. Significant values are calculated against the untreated group (^*∗*^*p* < 0.05).

The antioxidant activities in this study were determined using several assays. A significant (*p* < 0.05) increment in the antioxidant activity can be seen from the increase in FRAP and SOD levels and decreased NO and MDA levels. From Figure 2, a significant (*p* < 0.05) increment of FRAP level was exhibited by pineapple vinegar treatments. On the contrary, pineapple vinegar treatment exhibited a significant (*p* < 0.05) reduction of MDA and NO levels with high-concentration pineapple vinegar gave the most significant (*p* < 0.05) results as compared to other treatment groups. However, no significant (*p* > 0.05) effect was observed in the SOD enzyme activity across the tested groups.

### 3.4. Pineapple Vinegar Regulates Obesity and Inflammatory-Related Genes

**Figure 3:**
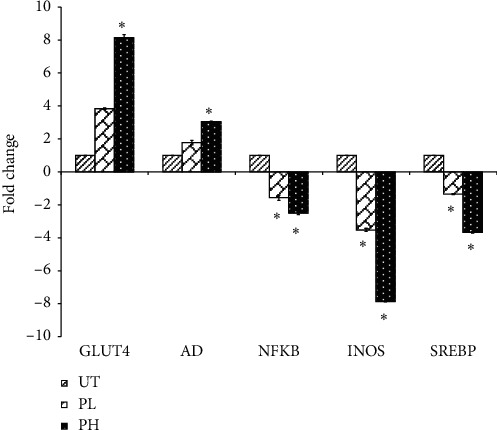
qPCR analyses on inflammation- and obesity-related genes of untreated (UT), 0.08 mL/kg BW pineapple vinegar (PL), and 1 mL/kg BW pineapple vinegar (PH). The data presented are represented as mean ± SD of biological replicate of mice from the same treatment group. Significant values are calculated against the untreated group (^*∗*^*p* < 0.05).

qRT-PCR was used to analyze the expression profiles of glucose transporter protein 4 (GLUT4), lipoprotein lipase (LPL), adiponectin (AD), nuclear factor kappa-light-chain-enhancer of activated B cells (NF-*κβ*), inducible nitric oxide synthase (iNOS), and sterol regulatory element-binding protein (SREBP) genes in the gonadal fat pad of control and vinegar-treated mice.  Figure 3 shows the mRNAs for the genes encoding GLUT4, LPL, AD, NF-*κβ*, iNOS, and SREBP. The highest upregulation of GLUT4 (∼7 folds), LPL (∼5 folds), and AD (∼2 folds) was associated with the highest downregulation of NF-*κβ* (∼2 folds), INOS (∼7 folds), and SREBP (∼4 folds) genes observed in the gonadal fat pad of mice treated with a high concentration of pineapple vinegar.

### 3.5. Pineapple Vinegar Regulates Adipokine Expression

**Figure 4:**
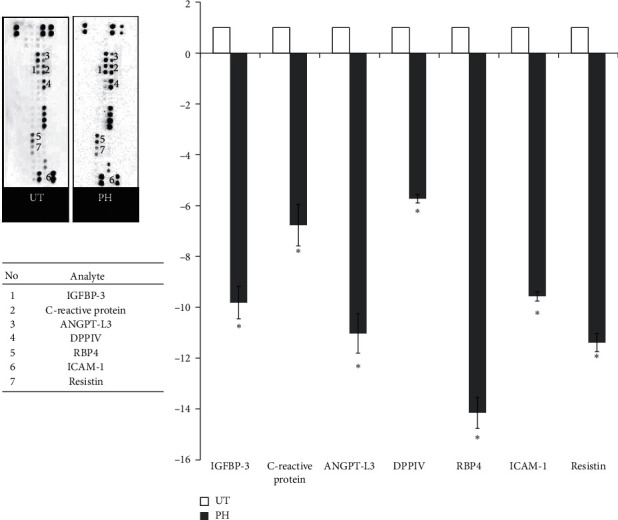
Adipokine proteome profiler analysis. UT = untreated group; PH = 1 mL/kg BW pineapple vinegar group. The data presented are represented as mean ± SD of biological replicate of mice from the same treatment group. Significant values are calculated against the untreated group (^*∗*^*p* < 0.05).

The quantitative changes in the adipokine expression were analyzed with adipokine proteome profiler, and the downregulation of all adipokines was observed in the gonadal fat pad of mice treated with high concentration pineapple vinegar (Figure 4). The highest downregulation of adipokine expression was exhibited by RBP protein (∼15 folds) followed by resistin (∼13 folds), ANGPT-L3 (∼12 folds), IGFBP-3 (∼11 folds), ICAM-1 (∼11 folds), C-reactive protein (∼8 folds), and DPPIV (∼7 folds).

### 3.6. Pineapple Vinegar Alters Gut Microbiota

**Figure 5:**
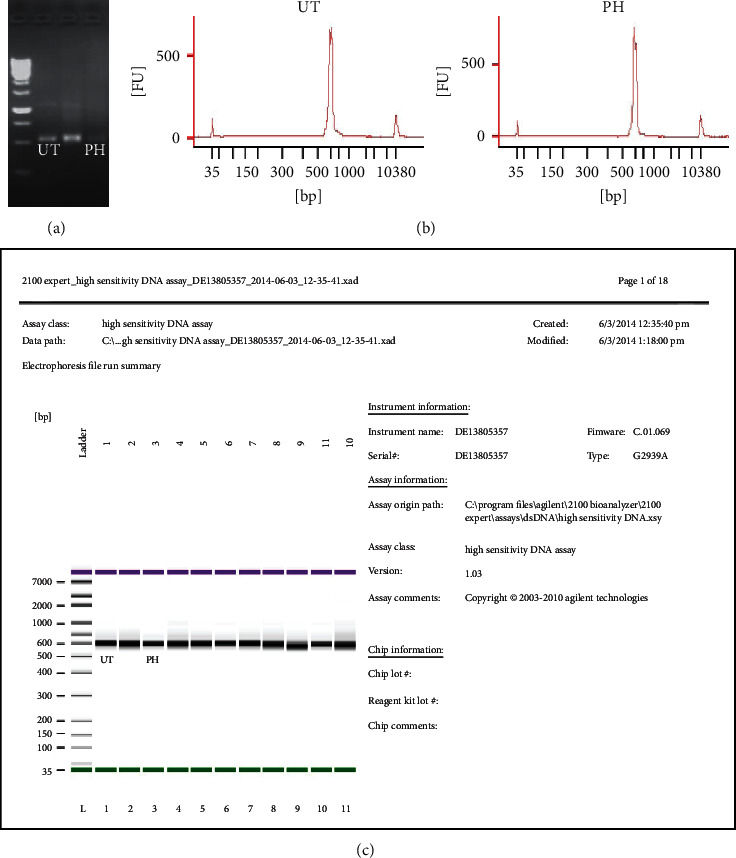
QC for metagenomic. (a) Gel electrophoresis of 16S DNA band for the first PCR run. (b, c) Bioanalyzer analysis of 16S rRNA band for 2^nd^ PCR. UT = untreated group; PH = 1 mL/kg BW pineapple vinegar group.

**Figure 6:**
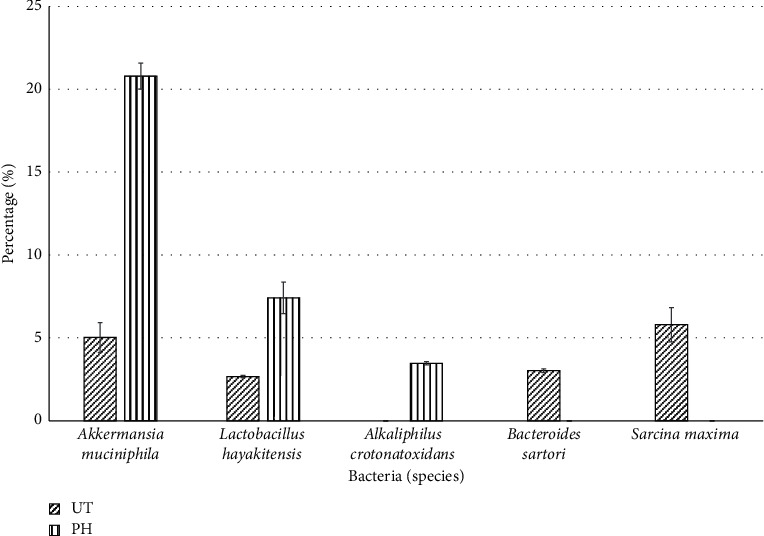
Metagenomic analysis. UT = untreated group; PH = 1 mL/kg BW pineapple vinegar group.

To evaluate the effect of pineapple vinegar on the alteration of gut microbiota population, NGS 16S rRNA metagenomic sequencing analysis was carried out.  Figure 5 shows the quality control (QC) results of the metagenomic analysis, while Figure 6 exhibited the shift in the top 5 microbial compositions in all treatment groups. As depicted in the figure, the relative compositions of *Akkermansia muciniphila* and *Lactobacillus hayakitensis* increased by ∼16 folds and ∼4 folds, respectively, in the pineapple vinegar group compared to the untreated group. The differences in the microbial communities can be seen in *Alkaliphilus crotonatoxidans*, *Bacteroides sartorii*, and *Sarcina maxima* species. From the data, only the pineapple treatment group shows the presence of *Alkaliphilus crotonatoxidans* (∼4 folds), while no *Bacteroides sartorii* and *Sarcina maxima* species were detected in the pineapple vinegar treatment.

## 4. Discussion

The obesity epidemic has become a serious health problem [3], and one of the factors that lead to this problem is the uncontrolled intake of dietary fat and sugar in daily life [42, 43]. Obesity is a systemic inflammatory response [44], and high levels of inflammation were found in many obese subjects [45, 46]. In the past years, studies offer the promise that several types of vinegar may help in reducing blood lipid, cholesterol, and fat accumulation and also improved the metabolic syndrome [32, 33, 36, 37, 46–48]. The study reported that the consumption of high-fat diet and obesity problems increased the cholesterol and triglyceride levels in the serum samples [49–51]. This is consistent with our data, where high levels of serum cholesterol and triglyceride were noticed in the untreated group.

Other than the elevation in the serum lipid profile and bodyweight, the consumption of a high-fat diet was also affiliated with an increase in oxidative stress [49–51]. MDA and NO are the by-products produced during oxidative stress [52]. Previous studies have proposed the effect of antioxidants in suppressing inflammation in the body [53, 54]. The antioxidant activities possessed by the pineapple vinegar may be one of the plausible factors contributing to the restoration of lipid serum and the regulation of all the genes and proteins in this study. Antioxidant plays a vital role in scavenging the free radicals formed from oxidative stress and restoring the level of cholesterol and triglyceride in the serum [55, 56]. From the overall results, the pineapple vinegar treatment significantly (*p* < 0.05) reduced the bodyweight and the ratio of the fat pad over bodyweight, improved the levels of serum cholesterol, triglyceride, and the HDL/LDL ratio, enhanced the FRAP activity, and reduced the level of MDA and NO as compared to the untreated group.

In the qRT-PCR quantification, our data revealed that the expressions of GLUT4, LPL, and AD were upregulated, while SREBP was downregulated in the gonadal fat pad of pineapple vinegar-treated mice. To note, most of the obese subjects have a higher insulin level [57]. GLUT4 plays an important role in transporting glucose when the insulin level is detected to be low in the body [58]. As the level of GLUT4 increased in the pineapple vinegar treatment group, it suggests that the treatment with pineapple vinegar may involve in the reduction of insulin level in the body. In addition, LPL is the gene involved in the hydrolysis of triglyceride [59]. In conjunction with the upregulation of LPL by the pineapple vinegar, the level of serum triglyceride was found to be lower as compared to the untreated group. Adiponectin, on the contrary, is responsible for regulating glucose and fatty acid oxidation in the body [60]. The study found that an increase in adiponectin levels in the body contributed to the weight loss process [61], and this is consistent with our data observed in the pineapple vinegar-treated mice. In contrast, SREBP involved in the lipogenesis and the synthesis of cholesterol, fatty acid [62], and triglyceride [63], and in this study, significant (*p* < 0.05) downregulation of SREBP was exhibited by the pineapple vinegar-treated mice.

**Figure 7:**
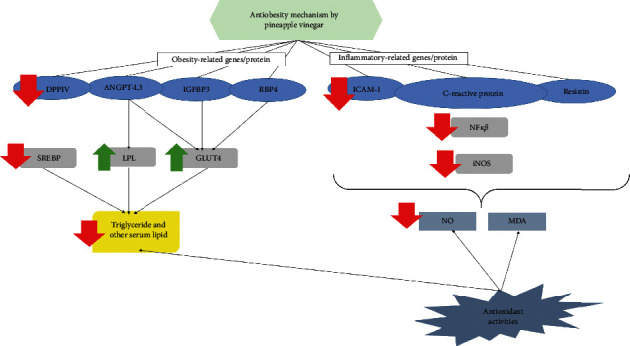
Molecular mechanism on the effect of pineapple vinegar as antiobesity.

Conversely, the positive correlations between the regulation of these genes with several adipokine proteins (IGFBP-3, ANGPT-L3, DPPIV, RBP 4, ICAM-1, and C-reactive protein) in the pineapple vinegar-treated mice are documented in Figure 7. The downregulation of IGFBP-3 was correlated with the inhibition of adipocyte proliferation through its capability to bind to IGF-1 and IGF-2 proteins. These IGF-1 and -2 proteins will subsequently suppress the IGF-1 receptor [64] and activate the insulin receptor [65]. The activation of the insulin receptor will then lead to an increase in GLUT4 expression [66]. These are consistent with our results shown in Figures 3 and 4. Besides IGFBP-3, angiopoietin-like 3 (ANGPT-L3) is another adipokine that is highly expressed in the liver and involves in the endothelial cell adhesion [67, 68]. The expression of ANGPT-L3 downregulated LPL and increased the level of plasma triglyceride [69–71]. Obesity was said to be associated with insulin resistance, and one of the factors that influence the insulin secretion is the blood glucose level and the expression of dipeptidyl-peptidase 4 (DPPIV) protein, which involves in the glucose metabolism [72, 73]. The presence of DPPIV inactivated incretin and glucagon-like peptide-1 (GLP-1) that is responsible for lowering the blood glucose, thus subsequently causing the elevation in blood glucose in the body [74, 75]. The downregulation of DPPIV in our data confirms the positive effect of pineapple vinegar in reducing obesity. Moreover, the regulation of retinol-binding protein 4 (RBP4) was also observed in the pineapple vinegar treatment group. RBP4 is involved in the insulin resistance process, where the upregulation of RBP4 was highly associated with obesity and diabetes type 2 problems [76]. The downregulation of RBP4 by pineapple vinegar also may be one of the factors contributing to the upregulation of GLUT4 expression in the qPCR result.

Obesity has been reported to be associated with acute inflammation, as the development of obesity promoted inflammation in the fat cells and adipose tissues [77, 78]. During the development of obesity, a high number of adipocytes were accumulated in the body, which promotes the onset of inflammation and thus increased the inflammatory-related genes, NF-kB, and iNOS [79, 80]. This obesity-induced inflammation will elevate the level of C-reactive protein, ICAM, and resistin, which subsequently increases the level of cholesterol in the blood [81–84]. In this study, a significant (*p* < 0.05) downregulation of these inflammation-related genes (NF-*κβ* and iNOS) and inflammation-related adipokine proteins (C-reactive protein, ICAM, and resistin) were recorded in the pineapple vinegar treatment groups, which was in line with the decreased level of NO, MDA, and serum cholesterol level.

Previous studies have described a strong correlation between obesity and the profile of gut microbiota [85, 86]. In this study, the faeces microbiota profile within groups was compared using NGS 16S metagenomics study [87]. Based on the result, the population of *Akkermansia muciniphila* and *Lactobacillus hayakitensis* was 4 and 1.6 times higher in the pineapple vinegar treatment group as compared to the untreated mice, respectively. *A. muciniphila* was found abundant in the treated obese mice [88], and polyphenol was recently reported as one of the factors contributing to the growth of *A. muciniphila* [89]. The abundance of *A. muciniphila* in the pineapple vinegar-treated mice possibly contributed by the rich polyphenolic contents in the pineapple vinegar (gallic, caffeic, benzoic, sinapic, vanillic, *β*-hydroxybenzoic, protocatechuic, and syringic acids) [41]. In addition, the increased *Lactobacillus hayakitensis* in the pineapple vinegar treatment group showed a positive effect on obesity treatment. Lactobacillus is a group of bacterium that maintains good gut health, and the association of lactobacillus bacteria and obesity has been long discovered [90].

Interestingly, we found that *Alkaliphilus crotonatoxidans* was present in the pineapple vinegar group but absent in the untreated group. However, the role of *A. crotonatoxidans* in gut health was still not well documented. Our data also showed that *Bacteroides satori* and *Sarcina maxima* were only present in the untreated group. Previous studies have found that the presence of Bacteroides family in the gut of children led to a higher risk of developing obesity in adulthood [91, 92]. On the contrary, the Sarcina family has been reported to contribute to the incidence of gastrointestinal symptoms such as bloating and stomach ulcers, which are commonly associated with obesity [93]. The absence of Sarcina and Bacteroides species in the pineapple treatment group showed the positive effect of pineapple vinegar treatment towards weight reduction through a healthy gut microbiota population. Overall, the metagenomic profile may represent viable interventions in obesity prevention as these factors strongly related to obesity and other metabolic diseases.

Comparing the effect of pineapple vinegar with coconut water vinegar [36], nipa vinegar, and synthetic vinegar [37], all vinegar was observed with significant (*p* < 0.05) body fat and bodyweight reduction. Besides, all treatments were also observed with reduced obesity-induced inflammation. Although all the fruits vinegar was reported with better effect than synthetic vinegar, different fruits vinegar still showed slight diverse in their bioactivities. More specifically, coconut water vinegar was reported to be the best effect to reduce bodyweight and fat pad/bodyweight ratio. On the contrary, pineapple vinegar was observed with the best effect to increase the HDL/LDL ratio and reduce related inflammation markers, including NO, inflammation-related genes, and adipokines. The superior anti-inflammatory effect may be contributed by the metabolites including gallic, caffeic, benzoic, sinapic, vanillic, *β*-hydroxybenzoic, protocatechuic, and syringic acids in the pineapple fruit [41, 94].

## 5. Conclusions

The antiobesity effects and the possible mechanism of pineapple vinegar were investigated in this study, and a summary of the overall mechanism is shown in Figure 7. Overall, pineapple vinegar regulated the expressions of several obesity-related genes and altered the gut microbiota to control inflammation and enhance the antioxidant level of mice with high-fat diet-induced obesity. Thus, pineapple vinegar can be used as a potential alternative functional food for obesity treatment.

## Figures and Tables

**Table 1:** Primer sequences.

*Target genes*	
AD	Forward: 5′-TCAGGAAGAGGAGGAGGA -3′
	Reverse: 5′-TCAGGAAGCACATCATACG -3′
SREBP	Forward: 5′-TCATCAACAACCAAGACAGT -3′
	Reverse: 5′-CCAGAGAAGCAGAAGAGAAG -3′
GLUT4	Forward: 5′-CTGCTTCTGGCTCTCACA -3′
	Reverse: 5′-AGGACATTGGACGCTCTC -3′
LPL	Forward: 5′-CGCTCCATTCATCTCTTCATTG -3′
	Reverse: 5′-GTTGCTTGCCATCCTCAGTC -3′
iNOS	Forward: 5′-GCACCGAGATTGGAGTTC-3′
	Reverse: 5′-GAGCACAGCCACATTGAT-3′
NF*κβ*	Forward: 5′-CATTCTGACCTTGCCTATCT-3′
	Reverse: 5′-CTGCTGTTCTGTCCATTCT-3′

*Reference genes*	
GAPDH	Forward: 5′-TTCCAGCCTTCCTTCTTG-3′
	Reverse: 5′-GGAGCCAGAGCAGTAATC-3′
ACTB	Forward: 5′-GAAGGTGGTGAAGCAGGCATC-3′
	Reverse: 5′-GAAGGTGGAAGAGTGGGAGTT-3′
HPRT	Forward: 5′-CGTGATTAGCGATGATGAAC-3′
	Reverse: 5′-AATGTAATCCAGCAGGTCAG-3′

**Table 2:** Percentage of the fat pad over bodyweight analysis of untreated (UT), 0.08 mL/kg BW pineapple vinegar (PL), and 1 mL/kg BW pineapple vinegar (PH).

Group	Final weight	Fat pad	Fat pad/BW (%)
UT	53.30 ± 0.50	2.19 ± 0.03	4.37 ± 0.06
PL	47.37 ± 3.33^*∗*^	1.62 ± 0.25	3.76 ± 0.58^*∗*^
PH	42.91 ± 0.73^*∗*^	1.48 ± 0.07	3.02 ± 0.14^*∗*^

The data presented are represented as mean ± SD of a biological replicate of mice from the same treatment group. Significant values are calculated against the untreated group (^*∗*^*p* < 0.05).

**Table 3:** Biochemistry profile of untreated (UT), 0.08 mL/kg BW pineapple vinegar (PL), and 1 mL/kg BW pineapple vinegar (PH).

Group	Cholesterol (mmol/L)	Triglycerol (mmol/L)	LDL (mmol/L)	HDL (mmol/L)	HDL/LDL
UT	5.21 ± 0.15	1.64 ± 0.01	0.75 ± 0.02	1.10 ± 0.15	1.47 ± 0.13
PL	3.34 ± 0.06^*∗*^	1.15 ± 0.01^*∗*^	1.39 ± 0.02	2.85 ± 0.09^*∗*^	2.05 ± 0.03^*∗*^
PH	2.43 ± 0.21^*∗*^	1.08 ± 0.05^*∗*^	0.61 ± 0.02	3.51 ± 0.15^*∗*^	5.75 ± 0.11^*∗*^

The data presented are represented as mean ± SD of a biological replica of mice from the same treatment group. Significant values are calculated against the untreated group (^*∗*^*p* < 0.05).

## Data Availability

The data used to support the findings of this study are included in the article.
